# Applying the Community Health Worker Coverage and Capacity Tool for Time-Use Modeling for Program Planning in Rwanda and Zanzibar

**DOI:** 10.9745/GHSP-D-20-00324

**Published:** 2021-03-15

**Authors:** Melanie Morrow, Eric Sarriot, Allyson R. Nelson, Felix Sayinzoga, Beatrice Mukamana, Evariste Kayitare, Halima Khamis, Omar Abdalla, William Winfrey

**Affiliations:** a ICF, Rockville, MD, USA.; b Save the Children, Washington DC, USA; now with Gavi, the Vaccine Alliance, Geneva, Switzerland.; c D-tree International, Zanzibar, Tanzania.; d Maternal Child and Community Health Division, Rwanda Biomedical Center, Ministry of Health, Kigali, Rwanda.; e Health Promotion Unit, Ministry of Health, Revolutionary Government of Zanzibar, Zanzibar, Tanzania.; f Avenir Health, Glastonbury, CT, USA.

## Abstract

Community health worker (CHW) programs are a critical component of health systems, notably in lower- and middle-income countries. However, when policy recommendations exceed what is feasible to implement, CHWs are overstretched by the volume of activities, implementation strength is diluted, and programs fail to produce promised outcomes. To counteract this, we developed a time-use modeling tool—the CHW Coverage and Capacity (C3) Tool—and used it with government partners in Rwanda and Zanzibar to address common policy questions related to CHW needs, coverage, and time optimization.

In Rwanda, the C3 Tool was used to analyze 2 well-established cadres of CHWs and 1 new one. The well-established CHW cadres were within a “manageable” workload range whereas the new cadre was projected to achieve less than half of assigned activities. This is informing ongoing changes to the CHWs' scopes of work. In Zanzibar, the C3 Tool was used to update the national community health strategy to include community health volunteers (CHVs) for the first time and determine how many CHVs were needed. The tool projected that 2,200 CHVs could achieve approximately 90% coverage of all defined services. Based on these figures, Zanzibar updated its national community health strategy, which officially launched in February 2020.

We discuss lessons from these 2 experiences. Translating analysis into decision making depends not only on the programmatic will and motivation of governments but also on finding opportune timing for when policy and program processes allow for optimization of CHW investments. Further research is needed but our experience supports the value of a modeling tool to ground program plans within estimated constraints on CHW time.

## Abstract (precis)

The C3 Tool supports community health worker (CHW) program planning by making tradeoffs apparent between human resources and the services to be provided at varying levels of population coverage. Governments in Rwanda and Zanzibar used the tool, respectively, to optimize CHW time allocation and to estimate how many CHWs were needed to meet universal health coverage goals.

## Teaser

**Key Findings:**

- Governments in Rwanda and Zanzibar used the Community Health Worker Coverage and Capacity (C3) Tool to optimize community health worker (CHW) time allocation and to estimate how many CHWs were needed to meet universal health coverage goals, respectively.
- In Rwanda, 2 well-established CHW cadres were within a “manageable” workload range, whereas a new cadre was projected to achieve less than half of assigned activities.
- In Zanzibar, the model projected that 2,200 community health volunteers could achieve approximately 90% coverage of all defined services. Based on these figures, Zanzibar updated its national community health strategy, which officially launched in February 2020.

**Key Implications:**

- Policy makers can use the tool to check the feasibility of existing or new CHW policy and strategy with respect to CHW numbers, workload, and population coverage of interventions.
- Program managers may find the tool useful to establish reasonable expectations for CHW contributions within existing policies and strategies, helping managers to make more realistic plans. Iterative use of the tool supports CHW strategy refinement. Used collaboratively, it can help with building consensus around decisions.

Résumé en français à la fin de l'article.

## BACKGROUND

The provision of sufficient human resources is a critical challenge for health systems in low- and middle-income countries (LMICs). Community health workers (CHWs)^1^ are recognized as a value-added workforce in the health system architecture, essential to the revitalization of primary health care and progress toward universal health coverage.^2^^–^^6^ Global research findings and national policy forums have progressively identified “what works,” as policies are established, task shifting and task sharing are expanded, and activities assigned to CHWs increase.

Many in the global health community fear that programs risk asking too much of their CHWs, in terms of tasks and burden of work, and will correspondingly underperform in achieving effective coverage. Past studies and landscape analyses of CHW programs in LMICs show a great diversity of status (paid, unpaid volunteer, part-time, or near full-time) and roles (home visits, community mobilization, social and behavior change communication, and preventive or curative clinical services).^2^^,^^4^ Time dedicated to ancillary activities (administration, training, supervision, and travel) also shows substantial variability.^7^^–^^9^ An article on 29 national CHW program case studies by Perry et al. provides a remarkable qualitative and quantitative update on this diversity.^10^

Decision makers develop policies for CHW roles based on each country's precedent, the emerging evidence about what services CHWs can deliver effectively, and since early 2019, the World Health Organization (WHO) guideline.^1^^,^^4^^,^^11^^–^^13^ In terms of operational variables (time use, population targets per CHW, etc.) evidence on the effectiveness of an optimal population size for CHWs, according to the Global Health Worker Alliance's systematic review, was both “limited” and “ambiguous.”^11^ The review concluded that the:

*estimation for an optimal population size [per CHW] would depend on various factors including the number and type of services and the actual time required for CHWs to complete their assigned tasks.*

This calls for tools to support country programs, and new tools have become available, notably to look at the cost issues.^3^^,^^14^ However, operational planning guidance is limited and not always specific to CHW programs,^2^^,^^15^ with the risk of overwhelmed community health workforces unable to deliver on strategies.

This article does not aim to make the case for the essential value of CHWs and effective CHW programs, which we believe is made clearly elsewhere.^1^^,^^16^^,^^17^ Neither do we seek to discuss or promote more integrated versus more vertical or more specialized versus more multisectoral community health workforces. Those are essential questions, but our purpose is more fundamental, addressing a basic operational question “in the weeds” of design and implementation of CHW programs, under any configuration.

We aim to address a basic operational question “in the weeds” of design and implementation of CHW programs.

We write as facilitators and country policy and program leaders in assessing and optimizing the time allocation of national CHW cadres on essential tasks through a new modeling tool. We focus on the experience of the Rwanda Biomedical Center (RBC), the government's central health implementation agency under the Ministry of Health (MOH), with support from the U.S. Agency for International Development's (USAID) Maternal and Child Survival Program (MCSP) and the experience of the Zanzibar Ministry of Health (ZMOH) with support from D-tree International.

## CHW COVERAGE AND CAPACITY TOOL: DEVELOPMENT AND DESCRIPTION

MCSP developed the CHW Coverage and Capacity (C3) Tool in Microsoft Excel based on a review of the literature on CHW time use and performance, iterative prototyping, and “lab testing.” USAID prioritized the C3 Tool's development with MCSP as part of a broader agenda on institutionalizing community health^18^ that included tools and technical assistance to support optimization of CHW policy implementation.^19^ The proof of concept was demonstrated in 2015 with 2 district managers in mainland Tanzania, in consultation with CHWs and their supervisors. The C3 Tool showed an important difference between the coverage expectations of CHWs and the reality of time and travel constraints. The tool has been used in 5 country settings as part of testing and development, including now mature implementation in Rwanda and Zanzibar. The current version of the tool and accompanying user guide are publicly available.^20^^,^^21^

The C3 Tool is used to analyze the correspondence between the number and type of activities implemented by up to 6 different CHW cadres versus the time available to implement these activities, factoring in country-specific epidemiology and demography. The tool is flexible, with CHW activities defined by the user to reflect the context. An interactive policy screen provides graphs and tables, allowing decision makers to explore the implications of various combinations of intervention packages and CHW time allocation to address 3 types of programmatic questions:

1. Quantification of needs for CHWs given a set policy: given C3 input parameters, how many CHWs are needed to achieve targeted coverage?
2. Quantification of the expected coverage, given a set number of CHWs: how much of the prescribed activities can materially be carried out by a fixed number of CHWs given the time they have available? This provides the equivalent of an expected coverage rate for CHW-delivered interventions. The tool allows adjustment of the parameters for population target size, for example, if a proportion of services is expected to be delivered in a facility and CHWs are expected to cover services in “the last mile.” This coverage can be revised over time or based on differences in regional balance of needs between facility and community-based services.
3. Optimization, given the number and level of effort of CHWs: How do possible changes in prioritization of interventions or changes in strength of implementation (more or fewer home visits, more or fewer “integrated” activities) affect intervention coverage and demands on time? This can also inform differential regional deployment choices.

The C3 Tool is used to analyze the correspondence between the number and type of activities implemented by CHWs versus the time available to implement these activities.

### Country Selection and Implementation Context

The 2 countries self-selected and were the most recent sites of C3 Tool implementation, feeding into their own active decision-making process.

Rwanda's community health program is well-recognized for having contributed to the country's achievement of the Millennium Development Goals for health.^22^ It is described in detail elsewhere.^10^ The national CHW program started in 1995 and later expanded in 2005 to include 3 CHWs for every village nationwide: 1 female maternal health assistant (agents de santé maternelle [ASM]) tasked with maternal and newborn health, community-based family planning, and health promotion; and a male-female binôme (pair) jointly responsible for integrated community case management (iCCM) of malaria, pneumonia, and diarrhea in children; community-based family planning; nutrition, including monthly screening of children aged under 5 years; TB referral and treatment adherence; home-based management of malaria for adults; and health promotion. CHWs also report community health data and provide referrals. CHWs in Rwanda are volunteers but receive financial incentives linked to community performance-based financing and participation in CHW cooperatives. Rwanda's community health program was comprehensively evaluated in 2016; findings included uneven implementation of services and recommendation to account for variations in setting such as urban vs. rural, population density, and epidemiologic trends.^23^ In 2019, a fourth CHW specifically for health promotion (HP-CHW) was elected per village, with responsibilities including early childhood development (ECD) and nutrition, among others. As of this writing, the Rwanda community health program reports a total of 58,567 CHWs (15,361 ASMs, 29,314 binômes, and 13,892 HP-CHWs) serving a total population of 12.37 million people (2019 estimate)^24^ and the CHW program is under further revision. RBC/MOH chose to partner with MCSP to carry out the C3 Tool analysis for all 3 of the government's CHW cadres, with special interest in the new HP-CHW cadre, as it had been recruited but not trained and its scope of work was in flux.

In 2019, the ZMOH and the President's Office of Regional Administration, Local Government, and Special Departments decided to update Zanzibar's national community health strategy. The previous strategy (2011) relied on local health committees to manage community health activities but did not include a cadre of CHWs. Based on the success of smaller nongovernmental organization-led programs, the ZMOH decided to formally adopt a volunteer cadre of CHWs in support of universal health coverage goals in the revised strategy (2019). CHWs in Zanzibar are called community health volunteers (CHVs) and are remunerated with performance-based incentives. As most of the population lives within 5 km of a health facility, the ZMOH decided CHVs should provide only preventive and promotive reproductive, maternal, newborn and child health, nutrition, and ECD services. However, the specific interventions within the package of services they could provide, the frequency of household visitation, the desired coverage of each service, and the overall total number of CHVs needed to meet these targets without exceeding a maximum time commitment of 18 hours per week had yet to be defined. With technical assistance from D-tree International, the ZMOH used the C3 Tool to answer these key questions which would form the foundation of the CHV program structure in the updated national strategy. The C3 Tool and this article both use the term CHW generically except when referring to specific cadres, such as the CHVs in Zanzibar.

## METHOD

The C3 method is a decision-making process supported by an interactive modeling tool for defining priority program time-commitments of CHWs. Although there are iterations and back-and-forth in the method, depending on context, the C3 process follows 5 major steps (Table 1). Steps 3 and 4 rely on the C3 Tool in Excel.

**TABLE 1.** Summarizing C3 Steps and Timelines in Rwanda and Zanzibar

	Rwanda	Zanzibar
1. Engaging stakeholders	2017–2018: District level testing and preliminary conversations with national stakeholders April 2019: Confirmed commitment to applying Tool with RBC/MOH for analysis at national level	September 2018: Engaged with key decision makers for community health strategy (ZMOH, UNICEF, Save the Children)
2. Defining the questions	April, July 2019	October 2018
3. Modeling assumptions and inputting data in the C3 Tool	July 2019: MCSP-RBC/MOH in-country consultation	October–November 2018
4. Iterative testing of scenarios through the C3 Tool	July 2019: MCSP-RBC/MOH in-country consultation	December 2018–January 2019
5. Prioritizing and decision making	Ongoing by RBC/MOH	January–February 2019: Followed by minor revisions when coupled with Management Sciences for Health's Community Health Planning and Costing Tool costing data July 2019: Finalized revised community health strategy February 2020: Launched strategy at Annual Joint Health Sector Review meeting

Abbreviations: MCSP, Maternal and Child Survival Program; MOH, Ministry of Health; RBC, Rwanda Biomedical Center; UNICEF, United Nations Children's Fund; ZMOH, Zanzibar Ministry of Health.

### Engaging Stakeholders

The first step in implementation usually builds on prior partnership engagement with national or subnational structures. Before the C3 Tool can be used effectively to change policy or program implementation, government decision makers and supporting partners must recognize the need and value of exploring priority services for CHWs in comparison to effective time available and seek to find a solution for this operational and management issue that balances all requirements and strategy or program objectives.

In Rwanda, MCSP worked with the community health program at the district level to assess time use of different CHW cadres in 2017. This work ultimately led to carrying out a C3 exercise at the national level. The process was protracted to accommodate government planning timelines and interruptions related to a transition of leadership in the community health program, under the responsibility of RBC/MOH. MCSP was invited to send a team to Kigali to complete a C3 exercise in July 2019 as the Rwanda Community Health Program was adding a new CHW cadre for health promotion.

In Zanzibar, the community health strategy development committee became interested in the C3 process as part of its planning for strategy revision. A team was formed within the committee to work on modeling using C3 with the support of D-tree and subsequently to provide recommendations back to the larger committee. Community health strategy committee leadership and composition reflected both the ZMOH Health Promotion Unit responsible for community health activities and the ministry of local government (President's Office of Regional Administration, Local Government, and Special Departments) responsible for implementing health services and managing staff.

### Defining the Questions

The clear definition of research questions is an essential initial step in the process. As previously described, questions could pertain to number of CHWs needed, expected coverage given a set number of CHWs, and scenarios for optimizing parameters for coverage.

For Rwanda, the driving question was how much coverage the newly designated HP-CHWs could achieve with their number and tentative responsibilities. Given that there were already 2 types of CHWs widely available in communities—2 binômes and 1 ASM per village—this also allowed examination of what coverage was expected of these well-established cadres for their respective scopes of work. Additionally, the Rwanda community health program leadership wanted to examine the implications of known difference in malaria epidemiology between regions, with special concern for rural areas. This led to the selection of 2 regional typologies for the analysis (Table 2). Identification of optimization opportunities was the ultimate objective of the exercise.

**TABLE 2.** Rwanda Model Assumptions by Typology: Population and CHWs

	Rural Eastern: Higher Malaria Typology,^a^ No.	Rural Northern and Western: Lower Malaria Typology,^a^ No.
Total population^b^	2,600,814	2,178,695
Under-5 population	338,106	286,259
ASMs	3,794	3,456
Binômes	7,799	6,949
HP-CHWs	3,837	3,392
Population/all-CHWs	169	158

Abbreviations: ASMs, agents de santé maternelle; CHWs, community health workers; HP, health promotion.

a Typologies: (1) Rural Eastern: highest relative malaria incidence, included 5/6 districts in province with lowest population density, maximum 455/km^2^; and (2) Rural Northern and Western: lower malaria incidence, included districts with less than national median population density of 481/km^2^.

b Population figures based on 2012 census data.

As Zanzibar was embarking on the rollout of its new CHV scheme, the first question was about quantifying the need for these CHVs. As it became rapidly clear that programmatic needs would be greater than capacity, the question evolved toward priority setting and optimization.

### Modeling Assumptions and Inputting Data

**FIGURE 1:**
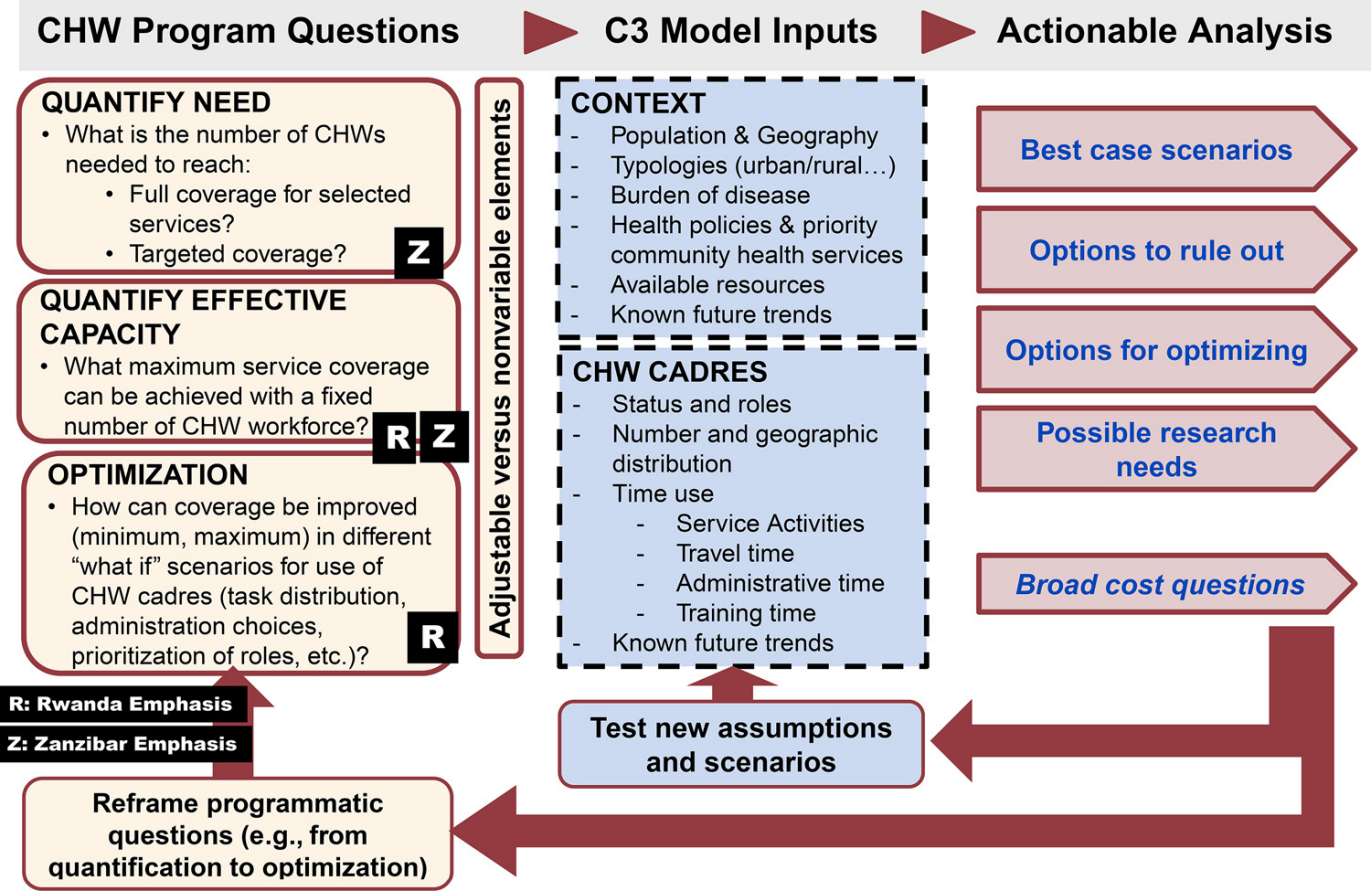
Iterative Steps of the Modeling Component of the C3 Process

This first step of the modeling exercise sometimes starts before substantial country engagement for known variables such as population size and number of existing CHWs. The default of C3 is to use national demographic data originating from World Population Prospects.^25^ Figure 1 maps the main stages of the C3 modeling steps.

Second, each country must establish values for a given set of parameters, including the level of effort of CHWs and time spent on various activities (e.g., administration, travel, and training, versus primary health interventions). The country must also set values for the nature of policy-supported interventions (e.g., home visits, engagement of groups in the community, campaigns, and referrals and accompaniment), with each intervention having a definition of the population targets, frequency of contact, and estimated time allocation. The full list of variables in the C3 model is provided in the user's manual^20^ and in the tool itself.^21^

The C3 Tool seeks to support management decisions that often must be made with imperfect data. Some variables have known and recognized estimates (national population and demographic distribution, CHW number). When the exercise focuses on a specific region, a first level of approximation enters in, for example applying or revising the national demographic profile to the region. Other variables have far less certainty, from the most basic information on time spent on work, travel times, even time spent on administration. When trusted data are not available, the facilitation team must engage with decision makers in making acceptable “reasonable assumptions.” Consultations with CHWs themselves and their supervisors can narrow down assumptions on travel and administrative work time: “at a maximum, time for activity X is not more than …” and “it is not less than ….” This is unsatisfactory from a research perspective, but par for the course for managers. Developing these assumptions also provides an opportunity for managers to become explicitly aware of management blind spots and increase the demand for follow-up assessments.

For Rwanda, data sources included national documents like the Community Health National Strategic Plan 2013–2018, the Comprehensive Evaluation of the Community Health Program: Final Report (2016), and the Demographic and Health Survey (2015); unpublished government statistics, and data from prior MCSP subnational intervention and engagement with CHWs.

The exercise in Rwanda, requiring differentiation between relatively higher and lower malaria prevalence regions, brought to light the limitations in translating malaria prevalence data into incidence rates, and consequently need for iCCM and adult home-based management services.^26^^–^^28^ We used prevalence rates and service statistics as benchmarks to establish that binômes from the Eastern province would need to see approximately 13 times the number of children seen in the Northern and Western provinces for malaria. In Zanzibar, the demographic parameters were adjusted to be specific to Zanzibar, rather than Tanzania as a whole.

In Zanzibar, this step and the following step overlapped closely. Population (1,579,849 total; 268,574 children under 5) and some demographic information in the tool was edited to reflect Zanzibar-specific unpublished 2018 data provided by the Office of Chief Government Statistician-Zanzibar. Time was spent specifying the package of services and specific interventions CHVs would provide, the methodology through which they would provide these (i.e., group counseling or household visits), the amount of time each integrated visit or session would take, the frequency of visits needed to have the desired impact on health outcomes or behaviors, and the population in need. The facilitation team consulted various units within the ZMOH to determine the frequency of campaigns and other ad hoc CHV activities. The delivery mechanism, frequency, and time was based on experience from previous programs and expert opinion on what each visit would look like when delivered with integrated content as specified in the package of services.

### Iterative Testing of Scenarios

This step is both the shortest and the most critical in the C3 process. Testing can take the format of a workshop or iterative checks with decision makers followed by adaptation of the model by the facilitation team. During these iterative checks, decision makers are given the first findings of the modeling exercise that address their questions, can ask “what if” questions, and can be encouraged to question the prioritization of tasks for CHW cadres. Facilitation requires both the ability to “play” with the model, as well as an understanding of community health programs, standards of evidence for proposed interventions, and practical programmatic sense.

In Rwanda, based on first findings, prioritized activities for HP-CHWs were immediately developed in consultation between Rwanda community health program leadership and the facilitation team. In Zanzibar, the package of services was defined by the community health strategy committee, but some interventions were designated as core interventions and others as add-on packages for a later time. The balance of group counseling versus household visitation was intensively discussed. Based on first findings, the facilitation team then sought ZMOH and community health strategy committee members' ideas for various scenarios, in particular, to determine financial and managerial resource availability to support a certain number of CHVs and reasonability of workload in terms of hours per week the CHVs could dedicate to their community health activities and the number of households they could each manage. Illustratively, TB direct-observation of treatment activities represented a disproportionate amount of the CHVs' time. They were deprioritized in later scenarios to maintain adequate coverage of reproductive, maternal, newborn and child health, nutrition, and ECD interventions.

In both settings, the facilitation teams provided national leaders with a summary of the iterative steps, findings, decisions, and a final suggested scenario submitted for national program and policy decisions.

### Final Prioritizing and Decision Making

The last step of the C3 process belongs to the policy and programmatic spheres of national and sometimes subnational leaders. It is highly contextual and not prescribed by the C3 process itself.

## FINDINGS

### Rwanda

**FIGURE 2:**
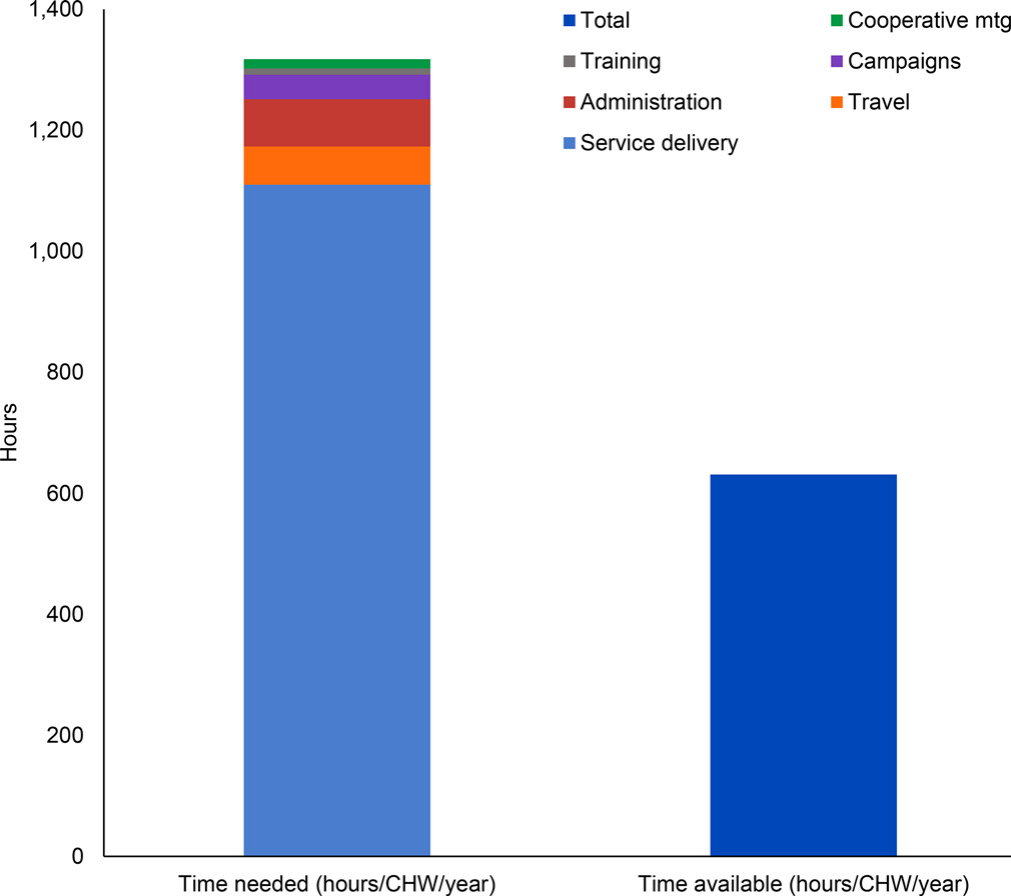
Graph Generated by C3 Tool of Annual Time Needed Versus Time Available per Health Promotion CHW, Default Scenario, Eastern Rural Typology, Rwanda Abbreviation: CHW, community health worker.

The team set out to determine if expectations were realistic or could be optimized for existing CHWs, assigned 4 per village, with special interest in the new CHW for health promotion. We found that the 2 CHW cadres with long-term management history (ASMs and binômes) could reach reasonable coverage (both more than 95%) based on the estimated workload required to achieve all their tasks whereas the new cadre tasked with health promotion was overloaded as modeled, projected to achieve under 50% of assigned workload. This was true for both typologies, with only minor differences. The large discrepancy for HP-CHWs between time needed verses time available to complete assigned tasks is illustrated in Figure 2. It shows that annually, a total in excess of 1,300 hours would be required per HP-CHW to carry out the services assigned in contrast to the 632 hours available.

In Rwanda, the tool showed that 2 CHW cadres could reach more than 95% coverage based on their workload, but a new cadre was projected to achieve less than 50% coverage.

Within service delivery, the tool identified that the most time-consuming activities assigned to HP-CHWs all involved home visits (behavior change communication targeting malnourished children; ECD visits for priority children; and insecticide-treated net use checks), followed by village-level hygiene club meetings, community cooking demonstrations, growth monitoring and promotion, additional follow-up with malnourished children, and group activities for ECD. Although making home visits multipurpose could improve efficiency, at least in theory, it was clear that the scope of work for the new HP-CHWs required further refinement. A second model or scenario reducing HP-CHW activities was created to align “priority intervention” time requirements to available time and shared with community health program leadership.

Rwanda subsequently took a different path through a more comprehensive reform of the CHW program. The current plan is to train all 4 CHWs per village in the same integrated package. This work is in progress and the design has not yet been modeled or tested, though building on the models from 2019 would expedite the exercise. RBC/MOH aims for efficiencies by task sharing, including greater potential for multipurpose home visits, and by digitizing reporting.

### Zanzibar

**FIGURE 3:**
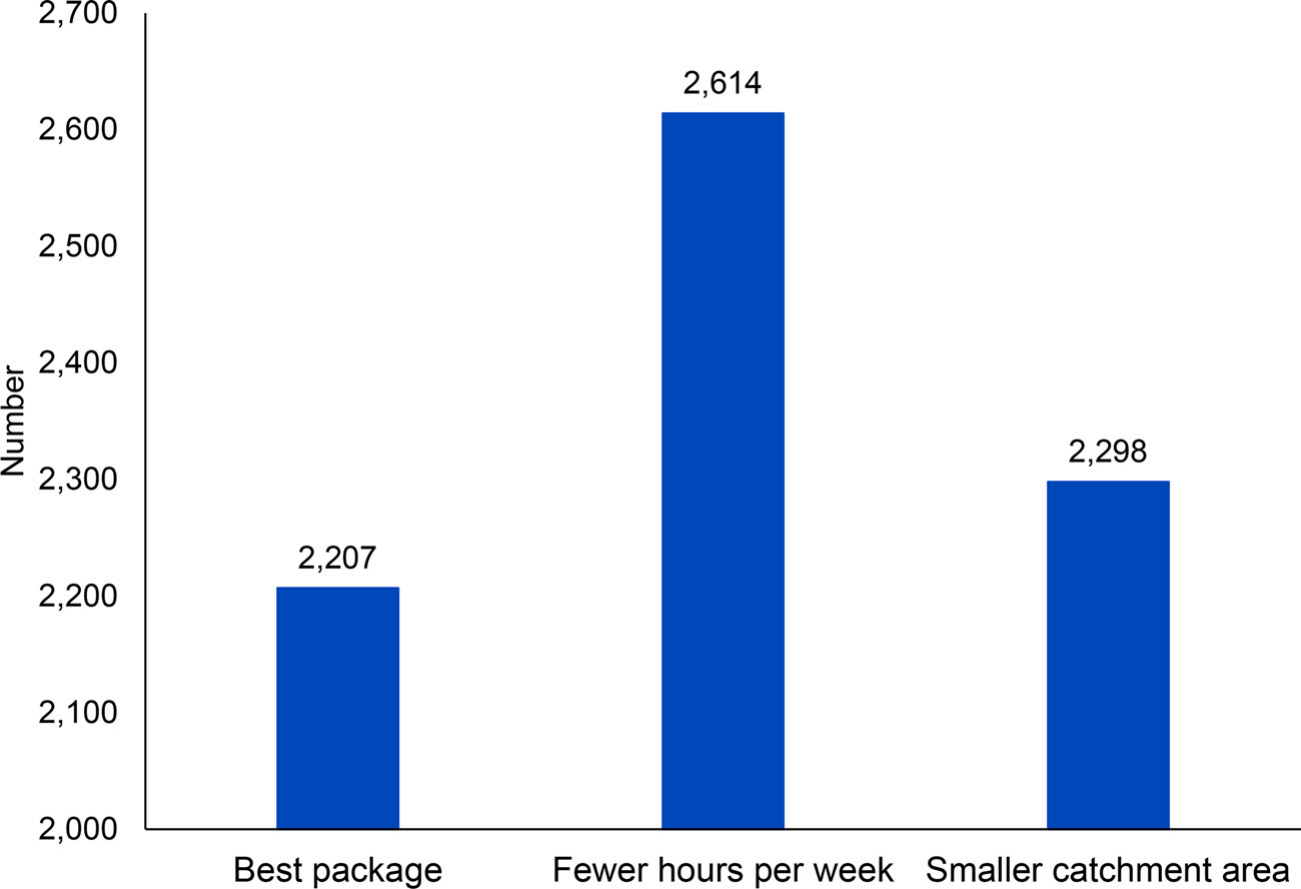
Number of Community Health Volunteers Needed in Zanzibar to Carry Out All Activities, as Calculated Using the C3 Tool

Through the iterative process previously described, the ZMOH determined that 2,200 CHVs, each serving a population of approximately 720 persons from 135 households, working on average 18 hours per week including travel time, should be able to reach 90% coverage of nearly all essential reproductive, maternal, newborn and child health, nutrition, and ECD interventions through a combination of household visits and group counseling sessions (Figure 3.) The top 5 activities CHVs would spend their time on included educational group counseling sessions, integrated child health visits, and extra time on identifying and coaching caregivers on threats to optimal child development. These were acceptable to stakeholders as the top priorities for CHVs' effort, which facilitated the final decision making to accept the proposed C3 scenario. Use of the C3 Tool also led to discussions about how to reduce CHV travel time (occupying 1/3 of available time) and adequacy of allowances for transport.

The Zanzibar community health strategy revision committee used the C3 modeling data to directly inform CHV numbers and services included in the new Community Health Strategy (2019–2025), formally launched in February 2020. In addition, the ZMOH with support from partners used the most promising scenario from C3 to conduct a separate costing exercise for the National CHV Program using United Nations Children's Fund/Management Sciences for Health's Community Health Planning and Costing Tool,^14^ confirming financial feasibility at national scale. Decisions in Zanzibar prioritized goals to achieve universal coverage of a service package that could be delivered within cost limitations.

In Zanzibar, the C3 Tool provided modeling data that informed CHV numbers and services that were included in the new community health strategy.

### Time Investment and Related Considerations for Tool Use

The time investment required to apply the C3 Tool varies by context and application. Variables include the complexity of the analyses to be undertaken and availability of supporting data, as well as factors such as the extent to which stakeholders are already organized around common purposes that tool use can both leverage and enhance. Using Zanzibar as an example, the process to collect and input data into the tool took approximately 2–3 weeks, consisting mostly of gathering existing statistics on demographics, health burden, and population in need, and then a second phase to estimate programmatic inputs such as length of visits, travel and administrative time, and frequency of campaigns. The Zanzibar team then took another 2–3 weeks to review content with stakeholders during multiple technical team meetings to validate assumptions and make revisions with wider ZMOH participation. National program managers used the tool to revise policy inputs such as the amount of time CHVs were expected to work per week and minimum acceptable levels of intervention coverage, and to remove some interventions that far exceeded CHVs' time available. These activities punctuated and fit well within the longer process for national strategy development and approval. Although the additional costs of using C3 were not measured, they were contained by leveraging existing partnerships and working groups in relatively small gatherings for consultations. The use of a technical partner who was familiar with the tool facilitating the information gathering, review, and revisions was effective and efficient.

## DISCUSSION

### Value of C3 for “Operational Realism”

The literature on time allocation, workload, and CHW productivity is sparse and inconclusive from a public health standard of evidence paradigm,^11^ making it difficult to answer questions about how many workers will be needed on average to deliver interventions using acceptable standard practice. The variation in contexts, history, and demand for services suggests that the value of **global** evidence may be to set limits on expectations and to propose broad guidelines for contextual adaptation of CHW programs.^1^^,^^4^

In Zanzibar, contextual adaptation went all the way to the local level. The ZMOH used the C3 Tool to estimate how many CHVs were needed on average per population for its policy, establishing an acceptable range within which local leaders defined individual CHV catchment areas that reflected variations in population density and requisite travel. According to unpublished national community health system data, Zanzibar has since found the total number of CHVs required in each district has closely aligned with what was modeled and that 90% of CHVs reported being on target for key performance indicators (e.g., number of household visits). Actual time spent for some services has been greater than in the C3 model, but time savings on travel, grouping visits by household, and geography may have balanced this and the overall positive coverage achieved. The Zanzibar team had anticipated that efficiencies could be found in effective management, including use of D-tree's digital application that all CHVs use to identify when children become “eligible” for a visit based on age and to group visits by household, contributing to confidence that the selected model scenario was within reason.

In Rwanda, the C3 exercise did not lead to an immediate policy change. However, the findings did call into question the ambitious demands put on the new community health worker cadre. Policy and CHW program adjustments are still being discussed under the Community Health Policy Review, involving a cross section of leadership and stakeholders from the Ministry of Health including RBC, as well as CHW supervisors and partners working in community health. An evolution of the complex CHW program architecture toward a single multipurpose/polyvalent volunteer cadre incentivized through performance-based financing is being deliberated, with a closer data-informed consideration of workforce size for manageable quality community health service provision and the need to mitigate workload imbalances. The process is ongoing.

CHW programs have shown tremendous value and, as mentioned previously, now benefit from WHO guidance,^1^ but their sustainability is of course resource-dependent even if they are cost-effective. If current efforts to resource and institutionalize them within national health system architectures are to continue, these programs will need to continue demonstrating added value, ultimately without dependence on development assistance mechanisms. Program impact will depend on multiple factors, but fundamentally 3 drivers, which we consider axiomatic.

CHW programs have shown tremendous value and proven to be cost-effective, but their sustainability is resource-dependent.

1. CHW interventions must be evidence-based.
2. They must be delivered in context with appropriate quality and strength of implementation at-scale.
3. To ensure this, they must be designed based on what we call “operational realism.”

The Google Dictionary and New Oxford Dictionary, respectively, define operational and realism as:

*relating to the routine functioning and activities of a business or organization*

and

*the quality or fact of representing a person, thing, or situation accurately or in a way that is true to life*.

We apply the terms here to mean that CHW program design and interventions must be feasible to implement under real-life conditions. This article focuses on 2 national examples, and we have implemented C3 exercises with 7 cadres of CHWs in 5 country settings (Egypt, Rwanda, Sierra Leone, mainland Tanzania, and Zanzibar) at different iterative stages of tool testing and development. For 5 of these 7 cadres, the C3 Tool documented expected coverage of services around or under 50%. This echoes the perceptions of many of our peers engaged in CHW programs and our assessment of the literature to-date that CHWs are frequently overtaxed. Building programs where the workforce cannot materially cover more than half of the targeted interventions and population fails the most basic sniff-test of operational realism.

Building programs where the workforce cannot materially cover more than half of the targeted interventions and population fails the most basic sniff-test of operational realism.

The process supported by the C3 Tool does not seek to guarantee impact, but it seeks to reduce guaranteed failure and unmanageability at local levels. It may serve as a useful guard against “empty scale-up,”^29^ by setting realistic limits within which local managers can develop solid and impactful plans.

### Complementarity With Other Tools

The C3 complements other tools that support community health and CHW program planning in the context of universal health coverage goals, each tool addressing 1 or more needs in the policy and planning process. We touch on several to highlight what the C3 contributes to this array. For example, the WHO Workload Indicators of Staffing Need tool^30^^,^^31^ supports generation of rich information on time use of and human resources needs for facility health workers but not for CHWs. Together C3 and WISN provide a more complete look at the time utilization of frontline health workers. New tools have also emerged or been adapted to estimate health workforce needs for surge and contact tracing in response to the coronavirus disease (COVID-19) pandemic.^32^^–^^34^

Previously mentioned, the Community Health Planning and Costing Tool allows users to calculate the costs of all elements of comprehensive community health services packages, including costs of start-up, training, and community-level service delivery, support, supervision, and management at all health system levels. The tool can be used to show program financing sources and gaps in current and future funding.^14^ In contrast, the niche of C3 is in time- and task-allocation and testing scenarios to guide optimization of the community health workforce investment. Once prioritized, the elements can be input into the Community Health Planning and Costing Tool for detailed analysis of resource needs and costing. In Zanzibar, the C3 Tool was used to identify limits of the policy, and then set the stage for use of the Community Health Planning and Costing Tool to inform costing, based on improved assumptions.

The CHW Assessment and Improvement Matrix (AIM): Updated Program Functionality Matrix^35^ and Lives Saved Tool,^36^ among other assessment and modeling tools,^19^ can help round out a plan. While both the CHW AIM and C3 support quality CHW program design and planning, the CHW AIM uses broader strokes across multiple program components without quantifying CHW needs or workload, the focus of C3. LiST can estimate the impact of an improved or expanded community health program by reducing morbidity and mortality but not the number of CHWs needed to implement said program.

### Diffusion of an Innovation?

The C3 is a modest innovation that provides a resource previously unavailable, allowing programmers to model scenarios for task prioritization and time allocation of mixed CHW workforces. The journey of development, testing, and implementation of C3 or any method to improve operational realism (which includes costing, budgeting, and functional analysis exercises mentioned above) goes beyond this article. However, we identify 2 types of conditions for the successful adoption of the tool and its full translation into actual programmatic decisions.

The first set of conditions lies within the national systems. Zanzibar and Rwanda are at different stages of development of their community health programs, but both are on a dynamic and intentional process of structuring and optimizing these programs. This perhaps positioned them to be early adopters.

The timing of the C3 process within the full decision-making process (policy or program) also matters. Zanzibar called for the exercise at a time of planning and intentionally sought to adjust plans to lessons from C3. Rwanda has a long history with CHW programs, multiple cadres, and has had multiple assessments and redesign steps. C3 came as 1 more analytical lens in a process of expanding the CHW workforce with a new additional cadre, while having had back-and-forth analyses about the need for the existing cadres, notably binômes who provide curative iCCM services. Different perspectives are at play in this redesign, and the current thinking has shifted to considering a universal distribution of tasks for all CHWs. Rwanda now has a template C3 model, to consider this new what-if scenario. Ultimately, the type of foundational operational thinking guided by C3 should be incorporated in all efforts to advance universal health coverage goals through CHW programs.

Given the number of countries currently reviewing, expanding, and trying to sustain CHW programs,^4^^,^^37^^–^^39^ it is surprising how often the syndrome of the “over-extended CHW” is bemoaned and yet so rarely addressed effectively.^40^^,^^41^ Humans and institutions behave by habit, and the habit of conducting reality checks has yet to be fully built into CHW programs. We can only conjecture that community health might remain just enough on the margins of traditional health systems planning and organizing that its core workforce requirements lack adequate attention and professionalism. If this is the case, we must question whether this is a question only for national health systems' leaders or whether it also relates to implicit biases in development assistance for health partners. And although community members and clients of community-based services may provide feedback on the availability, accessibility, and quality of services that they receive, elements such as time allocation, task distribution, and workforce size are variables generally beyond their appreciation, in the details of management.

Regardless, finding the right moment and right balance of national and local stakeholders to engage in C3-type processes will be key for the future. For example, in a prior country testing exercise carried out in a workshop, direct CHW supervisors voiced enthusiasm and renewed commitment to their work as an outcome of the workshop. They stated that they had a way to help prioritize the CHWs' work toward achievable objectives. Ongoing engagement with central program overseers suggested that other factors weighed heavily in defining priorities outside of operational feasibility. We were not set to study explicit versus implicit decision-making rules of policy makers as part of that exercise, but impressions echoed known tensions in formulating effective policies.

The second set of conditions for successful implementation lies with the facilitation team, which needs to combine 2 different skillsets: the ability to master an Excel modeling tool and the ability to keep the modeling tool as the servant of programmatic thinking. Prior exercises have shown that “gaming the model” can be a temptation in scenario testing. For example, gaming the model can mean integrating too many services within 1 timeframe for home visits of CHWs to achieve high coverage on the Excel outputs. Sound programmatic experience is required to challenge this temptation and to avoid confusing the “map” (the model) with the “territory” (real-time management).^45^

### More Research Is Needed

Although C3 is primarily a management and decision-making tool, it raises questions to be explored by research. There is the broad question of the dissemination of operational realism in CHW programs and decision making, the place of modeling tools themselves in this effort, and the role and benefit of engagement of decision makers at central versus decentralized levels, together or in different step configurations. Geographic and social context can vary substantially even within countries. How can central levels support better programming of human resources at the subnational level and ensure that field realities are reflected in implementation guidance?

The context of COVID-19 has only reemphasized questions about human resources management in community health programs, from task shifting and task sharing, to the institutionalization of resilient community health platforms able to respond adaptatively to emerging threats, while maintaining essential services. For C3 or any tool designed to support decision making, the questions will be about the evolution toward “portable,” easy-to-use tools in the toolbox of country program managers.

The COVID-19 pandemic has reemphasized questions about human resources management in community health programs, from task shifting to institutionalization.

### Limitations

This article describes how a tool and process can be used to address a structural, nuts-and-bolts, operational problem in CHW programming. Although the C3 has been tested or implemented at different scales in 3 other country contexts, we report on the 2 most mature exercises.

Some limitations of the tool may reflect misunderstandings about what it is. First, C3 does not seek to precisely measure how CHWs use their time. It seeks to compile and use available information and, when information is missing, build reasonable assumptions to guide decision making and generate rational plans. Although it may and will raise questions and suggest needed measurement efforts, its central purpose is to improve decisions at the time when policy makers and managers must make them. Consequently, we willingly admit there are wide confidence margins for C3 findings. Whether the modeled coverage of services to target populations is 45% or 55% is of limited importance if the goal is universal coverage. In simple words, “you can't get there from here.” In the case of Zanzibar, a scenario modeled to provide 90% coverage was considered “realistic enough” to guide policy, with expectations that close management could close the coverage gap through efficiencies, and that microconditions would be the main drivers of success. In Rwanda, the expected coverage for the new CHW cadre of all desired interventions clearly signaled that no amount of smart local planning and management could guarantee meaningful impact.

Developing reasonable assumptions can range from relatively simple—conducting group or individual discussions with a diverse set of CHWs in a region—to highly complex, as we discovered. In Rwanda, we tried to contrast the burden of effort placed on CHWs in a (relatively) higher malaria prevalence region compared to a low-malaria region. Translating available malaria prevalence estimates to incidence estimates, reflecting demand for services (both facility and community), proved impossible even with advice from experts in the field. An independent publication followed our effort and spells why this is so challenging.^46^ We were forced to look at service statistics and make extremely broad assumptions about how differences would play out at community level given facility-level reports. This speaks to the wide confidence margins previously mentioned but also demonstrates how data-oriented approaches to management can stimulate new and meaningful questions until they are glossed over. Given the low prevalence of malaria overall in Rwanda, the differential estimates did not play an important role in estimating the plausible achievable coverage of services by CHWs (binômes). However, this issue clearly flags an area where better surveillance and use of service statistics could improve decisions.

Last, we refined and adapted the tool in each implementation country, since time allocation, target groups, and intervention strategies can vary. This is inherent to the design and purpose of the tool (i.e., guide local decisions through the messiness of contextual variables), but it also limits the comparability of intermediary data from one country to the next, while preserving comparability of the outcome measures (human resources needs and expected coverage for prioritized evidence-based interventions).

## CONCLUSION

Operational realism must be central to the advancement of community health programs. The C3 Tool provides a way to analyze and understand time allocation challenges, which we believe are too often neglected and threaten the potential of CHW programs. We believe it is a promising tool that can serve the needs of political, policy, and program leaders to build more effective CHW programs at scale.

## Translation

**En Français**

Modélisation de l'utilisation du temps avec l'outil « C3 » pour l''analyse de la capacité et de la couverture des programmes d'agents de santé communautaires au Rwanda et à Zanzibar

**Message clé**

L'outil C3 soutient la planification des programmes d'ASC en permettant d'identifier de façon explicite les compromis à trouver entre capacité en ressources humaines et les services à fournir pour différents niveaux de couverture sanitaire. Les gouvernements du Rwanda et de Zanzibar ont utilisé l'outil, respectivement pour optimiser la répartition du temps des ASC et pour estimer le nombre d'ASC nécessaires pour atteindre des objectifs de couverture sanitaire universelle.

**Résumé**

Les programmes d'agents de santé communautaires (ASC) sont une composante essentielle des systèmes de santé, notamment dans les pays à revenu faible et intermédiaire. Cependant, lorsque les recommandations issues des politiques de santé vont au-delà de ce qui est opérationnellement réaliste, les ASCs sont surchargés, la force de mise en œuvre des interventions est diluée et les programmes ne peuvent pas fournir les résultats promis. Nous avons développé un outil de modélisation de l'utilisation du temps - l'outil d'analyse de la couverture des programmes ASC (C3: « CHW Coverage and Capacity tool ») - et l'avons adapté avec des partenaires gouvernementaux au Rwanda et à Zanzibar pour évaluer les besoins en ASC et/ou optimiser l'allocation du temps des ASC.

Au Rwanda, l'outil C3 a été utilisé pour analyser l'utilisation du temps de deux types d'ASCs bien établis d'une part, et d'un nouveau profile d'ASC, d'autre part. L'estimation de la charge de travail des deux types d'ASC déjà en place a été établie à un niveau « gérable », alors que la modélisation C3 suggérait que les nouveaux ASCs ne pourraient réaliser plus de la moitié des activités assignées. Ces résultats contribuent à informer un processus en cours d'adaptation du programme ASC dans son ensemble. A Zanzibar, l'outil C3 a été utilisé pour déterminer le nombre d'ASC nécessaires dans la mise à jour de la stratégie nationale de santé communautaire, qui devait pour la première fois inclure des ASCs. La modélisation a établi que 2 200 ASC seraient nécessaires pour atteindre une couverture d'environ 90% des services prioritaires. La stratégie nationale de santé communautaire de Zanzibar a été mise à jour sur la base de ces chiffres et officiellement lancée en février 2020.

Nous discutons des leçons de ces deux expériences. Transformer une analyse en prise de décision dépend non seulement de la volonté programmatique et de la motivation des gouvernements, mais aussi de l'existence de moments propices où processus politiques et programmatiques convergent et permettent l'optimisation des investissements dans les ASCs. Des recherches supplémentaires seront nécessaires, mais notre expérience confirme la valeur d'un outil de modélisation simple pour ancrer la planification et la programmation dans des limites réalistes pour l'utilisation du temps des ASCs.

**Peer Reviewed**
